# Brain imaging in patients with COVID-19: A systematic review

**DOI:** 10.1016/j.bbih.2021.100290

**Published:** 2021-07-02

**Authors:** Pablo Najt, Helen L. Richards, Dónal G. Fortune

**Affiliations:** a Department of Psychology, University of Limerick, Ireland; b Department of Clinical Psychology, Mercy University Hospital, Cork, Ireland

**Keywords:** SARS-CoV-2, COVID-19, CNS, Brain abnormalities, Neuroimaging

## Abstract

**Background:**

There is increasing evidence that SARS-CoV-2 (severe acute respiratory syndrome coronavirus 2) not only affects the respiratory tract but also influence the central nervous system (CNS), resulting in neurological symptoms such as loss of smell and taste. Growing literature indicates largely distributed brain alterations encompassing subcortical micro- and macro-bleeds, cerebral swelling and haemorrhage in gray and white matter tissue. A systematic review was performed to synthesise the potential evidence of the brain correlates of SARS-CoV-2.

**Methods:**

A literature search was conducted using electronic databases for studies reporting neuroimaging abnormalities in SARS-CoV-2 infected individuals. Identified case series, cohort studies, and case control studies on SARS-CoV-2 effects on the brain were critically appraised for methodological quality. A narrative synthesis of the findings from the included studies is presented.

**Results:**

Twenty-seven studies were included in the review, including 5 case series, 8 cohort studies and 14 case control studies. The findings revealed predominant involvement of the olfactory system with disruptions across four olfactory structures. Abnormalities also extended to the corpus callosum, cingulate cortex, and insula, jointly implicating the olfactory brain network.

**Conclusion:**

Alterations in olfactory areas, along with neighbouring brain regions, including prefrontal and limbic regions were associated to contraction of SARS-CoV-2. Viral infection could either trigger systemic reactions, or use the olfactory's unique anatomical organisation as an environmental entry zone to directly impact on the CNS.

## Highlights

• A spectrum of brain changes particularly at advanced stage relates to SARS-CoV-2
• Across severity phases SARS-CoV-2 affects olfactory structures
• Emerging abnormalities in COVID-19 is associated with the olfactory brain network

## 1 Introduction

In late 2019, the severe acute respiratory syndrome coronavirus 2 (SARS-CoV-2) emerged causing the new coronavirus disease (COVID-19). Viral infection resulted in more than 170 million laboratory-confirmed cases around the world, with over 3.5 million deaths as per 31st of May 2021 (Dong et al., 2020). Most patients with COVID-19 present mild to severe respiratory illness with symptoms such as fever, cough and shortness of breath, which might appear 2–14 days after exposure (Li et al., 2020). The majority of people with COVID-19 will generally require 2 weeks to recover from the respiratory tract symptoms (Hu et al., 2021). A substantial proportion of affected patients do not fully recover developing long-COVID (a postviral syndrome) despite testing negative for SARS-CoV-2 (Rubin, 2020). Although SARS-CoV-2 primarily targets the lungs, impact on multiple organs is increasingly been recognised (Gupta et al., 2020).

The brain is among the targets of SARS-CoV-2, resulting in neurological symptoms such as loss of smell and taste (Butowt and Bartheld, 2020). To date a number of systematic reviews have highlighted abnormalities on brain imaging as a major feature of COVID-19 (Choi et al., 2020; Moonis et al., 2020; Egbert et al., 2020). These reviews revealed neurological sequelae of COVID-19 including parenchymal brain abnormalities, subcortical micro- and macro-bleeds, cortico-subcortical swelling, and nonspecific deep white matter changes. These reviews contribute valuable data for identifying neurological diagnoses associated with COVID-19, however they focus on clinical radiologic issues without addressing specificity of impacted brain regions. Thus, it is important to address a number of remaining questions. This includes clarity over whether onset of these neurological signs during COVID-19 involve 1) direct viral invasion of the brain through the olfactory pathways or 2) viral invasion through the medullary cardiorespiratory center in the brainstem. This can certainly be elucidated by taking advantage of neuroimaging's ability to map implicated brain regions and their functional networks.

With the overall goal to elucidate the underpinning neuropathology of SARS-CoV-2, this systematic review examined brain changes following the virus infection. Special focus was devoted to identify structural and functional brain changes in the context of 1) severe and, 2) mild/recovery phases of COVID-19.

## 2 Methods

This systematic review was conducted in accordance with the Preferred Reporting Items for Systematic Reviews and Meta-Analyses (PRISMA) statement (Page et al., 2021) and prospectively registered with PROSPERO (CRD42021235796).

### 2.1 Search strategy

**Fig. 1:**
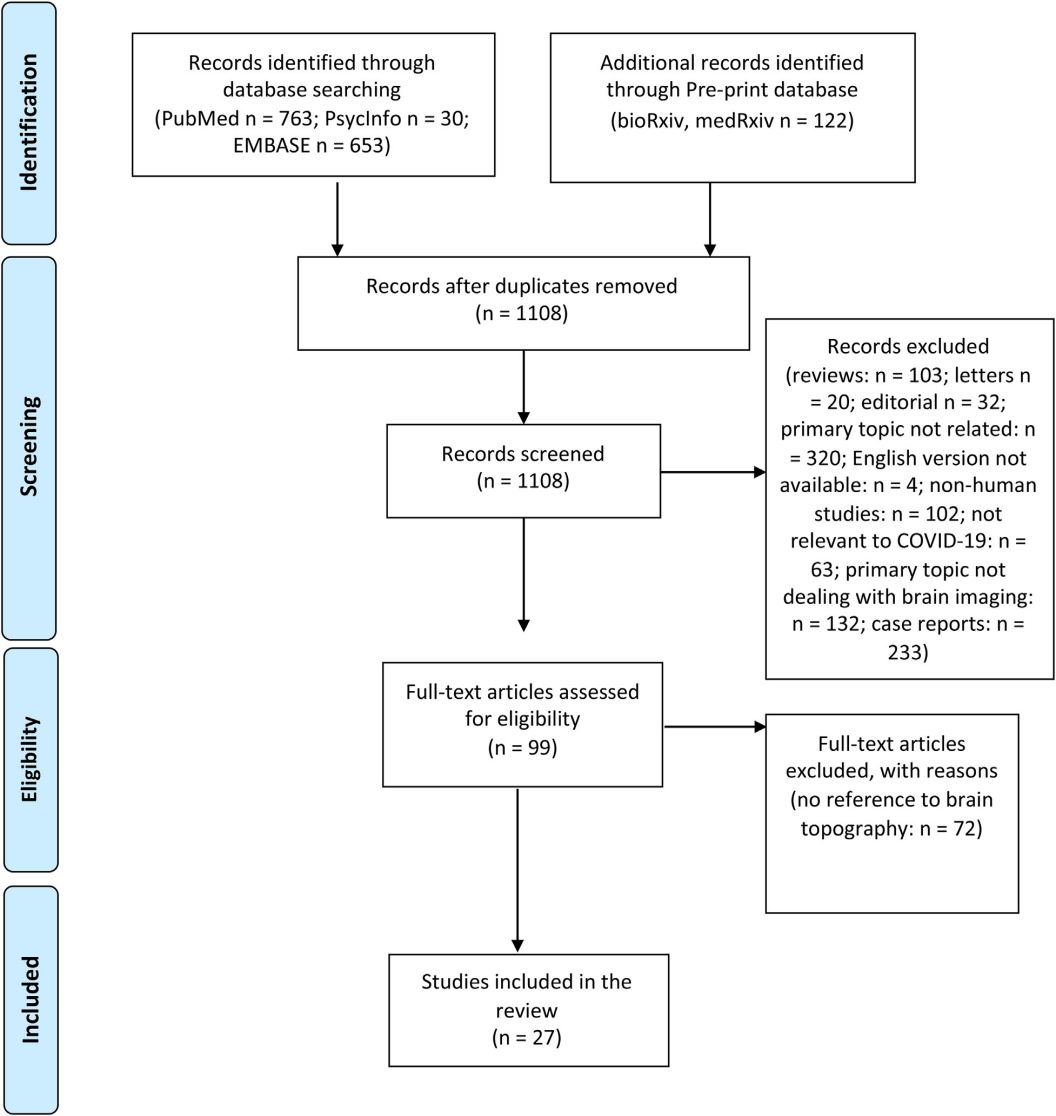
Prisma flow diagram.

PubMed, Embase, PsycInfo, bioRxiv, and medRxiv Database were searched for studies between January 1, 2021 and May 18, 2021. The following comprehensive search strategy was applied: (“SARS-CoV-2” OR “COVID-19” OR “Coronavirus”) AND (“neuroimaging” OR “brain imaging” OR “brain MRI” OR “MRI brain” OR “MRI head” OR “brain PET” OR “PET brain” OR “PET head” OR (“brain” AND “MRI” OR “PET”))”. PN conducted the literature search. Titles and abstracts were screened and full-text papers retrieved and reviewed. Reference lists from included articles and recent reviews (Choi et al., 2020; Moonis et al., 2020; Egbert et al., 2020) supplemented the literature search. Screening of titles and abstracts was undertaken by two independent reviewers (PN and DF) blinded to each other's’ decisions. Any disagreement and discrepancies were solved by discussion. The study selection process is outlined in Fig. 1.

### 2.2 Inclusion and exclusion criteria

Original contributions were included if they met the following criteria (PICOS). Population: (1) Focused on individuals with COVID-19 infection with a sample size greater than six individuals. Exposures: (2) Contraction of SARS-CoV-2 infection. Comparisons: (3) Individuals who have not been infected with COVID-19 as a control group. Outcomes: (4) Imaging findings from derived measures of brain structure/function (e.g., magnetic resonance imaging (MRI) or other modalities such as Positron Emission Tomography (PET)). Study design: Case control studies were included, but also cohort studies and case-series without a group control to aid further data on the final number of included studies. The exclusion criteria were as follows: (1) reviews, editorials, and letters not containing original data; (2) case reports or case series ​< ​7 patients; (3) studies not including sufficient details on imaging findings, i.e. brain localisation; (4) non-English language publications.

### 2.3 Data extraction and literature quality assessment

All articles identified from the search were imported into RefWorks and duplicates were electronically or manually removed. Two independent reviewers (PN and DF) applied the eligibility criteria and selected studies for inclusion in the systematic review. The studies were assessed according to the risk of bias through the JBI Critical Appraisal Checklist for Case Series (Munn et al., 2020), the JBI Critical Appraisal Checklist for Cohort Studies (Moola et al., 2020), and the JBI Critical Appraisal Checklist for Cross-sectional Studies (Moola et al., 2020), according to the study design. PN conducted the quality assessment. Two independent reviewers (PN and DF) carried data extraction from the studies selected for inclusion using a data extraction form to include the following categories: first author, year of publication, the PubMed identification number, setting, study design, number of patients with COVID-19, number of patients with neuroimaging, comorbidities, treatment support (i.e., ICU, ventilator), COVID-19 severity stage, control group, outcome measures, whether brain imaging was a primary focus of the study, and study findings.

### 2.4 Strategy for data synthesis

A formal narrative (descriptive) synthesis is presented. PN coded study information, and summarized and synthesised aggregate data, i.e., the results from each included study. Description of findings from all available studies included the results (i.e., whether or not these studies report brain changes associated to COVID-19), study design, and the sample size while taking especial note of comorbidities.

## 3 Results

The search identified a total of 99 brain neuroimaging studies on COVID-19 patients. Seventy-two of these studies evaluated neurologic signs without reference to brain topography and therefore, were not further considered. Consequently a total of 27 studies assessing brain changes following SARS-CoV-2 infection were included in the final review, comprising 5 case series, 8 cohort studies, and 14 case-control studies.

The included studies were deemed of sufficient methodological quality, according to the JBI Critical Appraisal Checklists (mean percentage scored points 95.7%), although some were of lower quality. The main missing quality components were as follows: Three case control studies differed significantly in their quality lacking ‘criteria for inclusion’ (Crunfli et al., 2021; Kas et al., 2021; Aragao et al., 2021; Silva et al., 2021), ‘consecutive inclusion of participants’ (Aragao et al., 2021), ‘valid methods used for identification of the condition’ (Aragao et al., 2021) ‘identified confounding factors’ (Crunfli et al., 2021; Raman et al., 2021; Guedj et al., 2021; Qin et al., 2021; Silva et al., 2021; Sollini et al., 2021; Strauss et al., 2020) and, ‘strategies to deal with confounding factors’ (Crunfli et al., 2021; Raman et al., 2021; Guedj et al., 2021; Qin et al., 2021; Silva et al., 2021; Sollini et al., 2021; Strauss et al., 2020). Two case series did not state whether ‘complete inclusion of participants’ was achieved (Fitsiori et al., 2020; Girardeau et al., 2020). Refer to Table 1 for a summary of quality of the included studies.

**Table 1:** Summarized quality assessment of the included articles.

Articles	Quality assessment
Sawlani et al.	10/10 (100.0%)^a^
Coolen et al.	10/10 (100.0%)^a^
Fitsiori et al.	6/8 (75.0%)^a^
Girardeau et al.	9/10 (90.0%)^a^
Conklin et al.	10/10 (100.0%)^b^
Dixon et al.	10/10 (100.0%)^b^
Freeman et al.	9/9 (100.0%)^b^
Chougar et al.	10/10 (100.0%)^b^
Kandemirli et al.	10/10 (100.0%)^b^
Klironomos et al.	10/10 (100.0%)^b^
Kremer et al.	10/10 (100.0%)^b^
Lin et al.	10/10 (100.0%)^b^
Aragao et al.	7/9 (77.7%)^a^
Lu et al.	6/6 (100.0%)^c^
Niesen et al.	8/8 (100.0%)^c^
Raman et al.	6/6 (100.0%)^c^
Crunfli et al.	4/6 (66.6%)^c^
Kas et al.	9/10 (90.0%)^c^
Blazhenets et al.	8/8 (100.0%)^c^
Donegani et al.	8/8 (100.0%)^c^
Duan et al.	8/8 (100.0%)^c^
Guedj et al.	6/6 (100.0%)^c^
Hosp et al.	8/8 (100.0%)^c^
Qin et al.	6/6 (100.0%)^c^
Silva et al.	5/6 (83.3%)^c^
Sollini et al.	6/6 (100.0%)^c^
Strauss et al.	6/6 (100.0%)^c^

a According to the Joanna Briggs Institute (JBI) Critical Appraisal Checklist for Case Series.

b According to the Joanna Briggs Institute (JBI) Critical Appraisal Checklist for Cohort Studies.

c According to the Joanna Briggs Institute (JBI) Critical Appraisal Checklist for Cross-sectional Studies.

Brain scans were assessed with essentially two contrasting approaches. Half of these studies (Fitsiori et al., 2020; Sawlani et al., 2020; Coolen et al., 2020; Chougar et al., 2020; Kremer et al., 2020; Kandemirli et al., 2020; Klironomos et al., 2020; Lin et al., 2020; Girardeau et al., 2020; Aragao et al., 2021; Conklin et al., 2021; Dixon et al., 2020; Freeman et al., 2021) performed visual inspection undertaken by qualified neuroradiologists and the other half (Crunfli et al., 2021; Lu et al., 2020; Niesen et al., 2021; Raman et al., 2021; Kas et al., 2021; Blazhenets et al., 2021; Donegani et al., 2021; Duan et al., 2021; Guedj et al., 2021; Hosp et al., 2021; Qin et al., 2021; Silva et al., 2021; Sollini et al., 2021; Strauss et al., 2020) implemented statistical techniques for examining differences in brain function and/or structure (see Table 2).

**Table 2:**
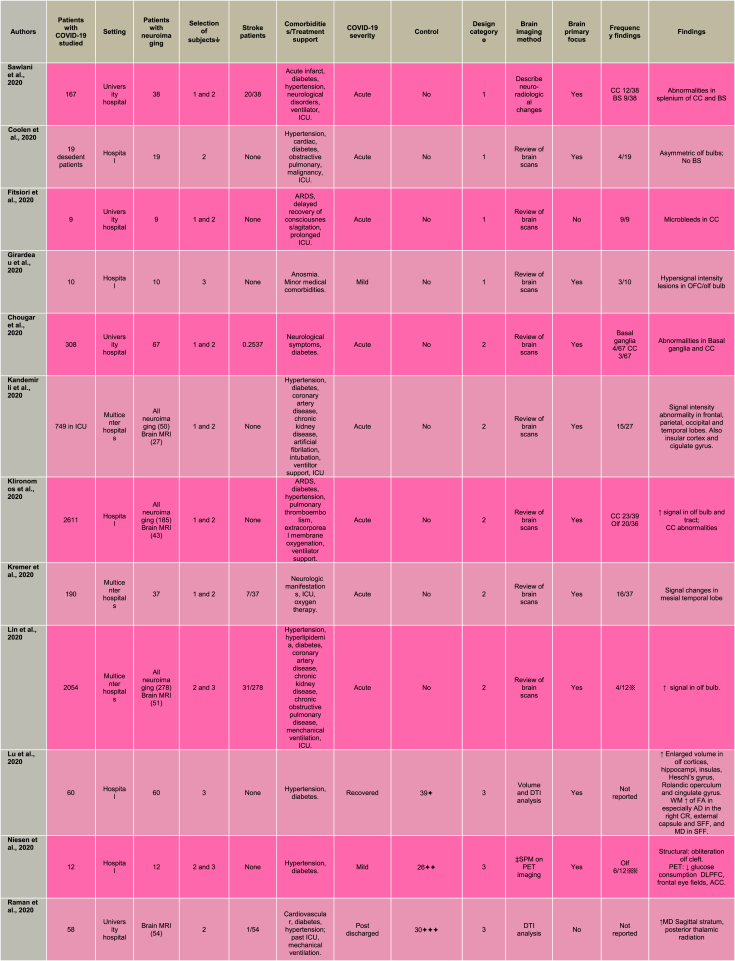

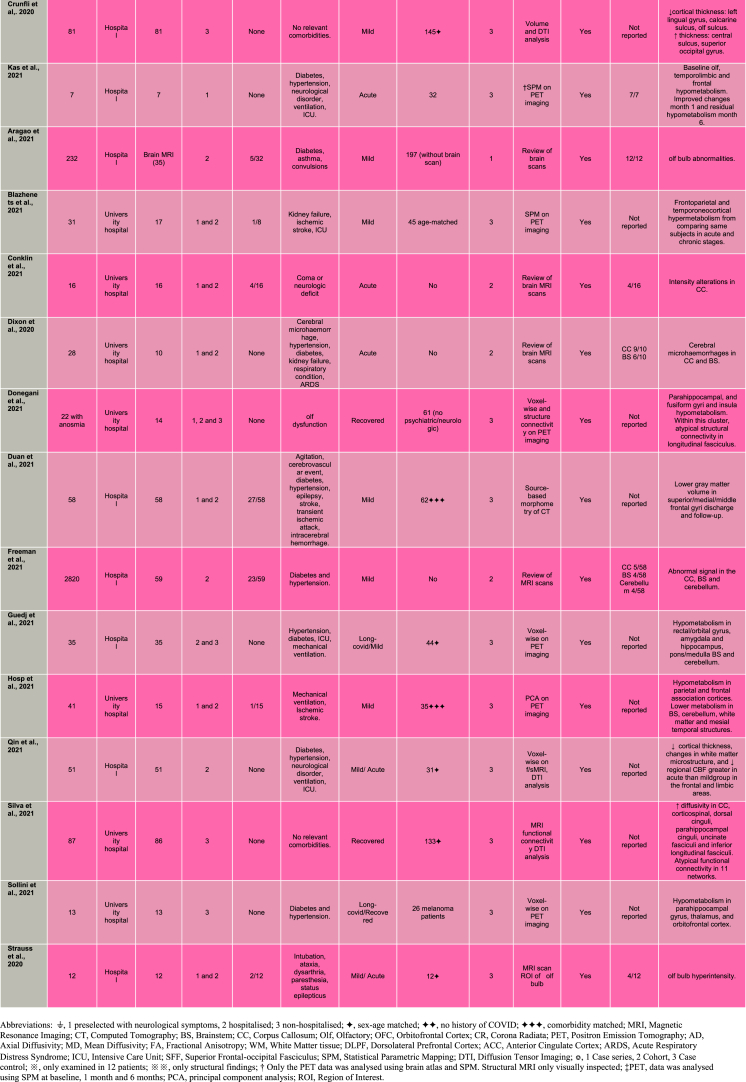
Neuroimaging findings on SARS-CoV-2.

### 3.1 Brain changes from acute effects of SARS-CoV-2

Acute stroke, a major neurologic complication of COVID-19 was reported by 12 out of the 27 included studies (Sawlani et al., 2020; Chougar et al., 2020; Kremer et al., 2020; Lin et al., 2020; Raman et al., 2020; Aragao et al., 2021; Blazhenets et al., 2021; Conklin et al., 2021; Duan et al., 2021; Freeman et al., 2021; Hosp et al., 2021; Strauss et al., 2020). The frequency of acute stroke ranged between 1.9 and 52 percent with a mean average of 22.7 percent across studies. Selection of participants included 14 studies that preselected for individuals with neurological symptoms, 8 studies selected hospitalised patients, and 5 studies selected non-hospitalised patients (see Table 2 for details).

Frequencies of brain alterations were reported by 16 of the 27 included studies. Alterations in the olfactory structures were present in 48 percent of cases that ranged between 2 and 100 percent across studies. Similarly, high incidence was identified in the corpus callosum, present in 48 percent of cases and ranging between 4 and 100 percent across studies. Frequencies of alterations in any other brain regions were reported by less than 3 studies as detailed in Table 2. Patients with COVID-19 presented with a spectrum of brain changes particularly at the advanced stage of infection. There was considerable variability in both the localisation and nature of brain abnormalities, including subcortical micro- and macro-bleeds, cerebral swelling and haemorrhage in gray and white matter tissue. Three case-series studies (Fitsiori et al., 2020; Sawlani et al., 2020; Coolen et al., 2020) investigated brain alterations at the severe phase of the infection implementing visual inspection of brain MRI scans undertaken by qualified neuro-radiologists. For example, a critically ill case-series of patients with acute respiratory distress syndrome (ARDS), delayed recovery of consciousness, and receiving prolonged intensive care unit (ICU) care reported microbleeds in the corpus callosum, internal capsule, middle cerebellar peduncles and subcortical white matter (Fitsiori et al., 2020). Moreover, white matter abnormalities involving the corpus callosum (splenium) and brainstem were seen in COVID-19 cases with a range of medical and neurological comorbidities (Sawlani et al., 2020). Finally, a post-mortem study on non-survivors of COVID-19 found parenchymal brain abnormalities including subcortical microbleeds, macrobleeds, cortico-subcortical swelling, non-specific white matter changes, and asymmetric olfactory bulbs (Coolen et al., 2020). Also this study explicitly examined the brainstem, however no alterations were detected in this brain area.

Acute SARS-CoV-2 infection and associated brain changes has also been investigated by cohort studies (Chougar et al., 2020; Kremer et al., 2020; Kandemirli et al., 2020; Klironomos et al., 2020; Lin et al., 2020; Dixon et al., 2020; Conklin et al., 2021). As with the case-series above, these cohort studies made use of radiological inspection of images, had similar sample sizes and retrospectively investigated MRI scans. Following the case-series findings, cohort studies reported abnormalities across a wide range of brain regions. Chougar et al. (2020) found signal and diffusion abnormalities in the corpus callosum and basal ganglia (substantia nigra, globus pallidus and striatonigral) in COVID-19 patients with some neurological symptoms. Kremer et al. (2020) reported signal abnormalities affecting the medial temporal lobe in patients with severe COVID-19 as the most frequent MRI finding. Furthermore, Kandemirli et al. (2020) found signal intensity abnormality in frontal (cingulate gyrus), parietal, occipital and temporal (insula) areas among a cohort of hospitalised COVID-19 patients with medical and neurological comorbidities receiving ICU care.

In contrast with the sparse findings above, four cohort MRI studies on hospitalised COVID-19 patients showed convergent patterns of abnormal signal intensity implicating the olfactory bulb and tract (Klironomos et al., 2020; Lin et al., 2020) and the corpus callosum (Klironomos et al., 2020; Dixon et al., 2020; Conklin et al., 2021). In a small cohort of COVID-19 patients with coma or neurological deficit, Conklin et al. (2021), found alteration in signal intensity in the corpus callosum. Another study reported corpus callosum and brainstem microhaemorrhages in COVID-19 patients (Dixon et al., 2020). Similarly, Klironomos and colleagues’ (2020) findings of disruption in the corpus callosum and increased signal in the olfactory bulb and tract were among the most frequent abnormalities in COVID-19 patients. Moreover, abnormally increased signal in the olfactory bulb, without volume changes, was shown in COVID-19 patients (Lin et al., 2020). More specifically focused on the question of brain olfactory integrity, Strauss et al. (2020) conducted a case control MRI study investigating signal alterations with a region of interest approach to calculate normalized values within the olfactory bulb. Notably, olfactory hyperintensity in COVID-19 patients was statistical significantly different relative to controls with anosmia.

Although some consistent brain abnormalities in olfactory areas and the corpus callosum have emerged, overall findings in acute COVID-19 patients are heterogeneous in nature (i.e., micro- and macro-bleeds, swelling, and haemorrhage) and type of tissue (parenchymal, cortical, subcortical and white matter); the extent of variability may be attributed to a synergistic effect from the characteristic medical and neurological comorbidities, and intensive treatment in these patients. The study of SARS-CoV-2-infected individuals with less severe manifestations may be a promising approach to investigate intrinsic effects of COVID-19 on the brain independent of severe comorbidities.

### 3.2 Localised brain repercussions of SARS-CoV-2 at mild or recovered phases

While influence of SARS-CoV-2 infection is possibly spreading throughout different brain areas, the olfactory system appears to be a consistent focus in mild and recovered cases. Two small MRI case series in individuals with mild COVID-19, among which one additionally focused on persistent loss of smell (Girardeau et al., 2020), using radiological inspection of brain MRI, found hypersignal intensity lesions in the olfactory bulbs (Girardeau et al., 2020; Aragao et al., 2021) and olfactory cortex (Girardeau et al., 2020). In a subset of COVID-19 patients presenting leukoencephalopathy (damage to white matter tissue), Freeman et al. (2021) found distribution patterns of abnormal MRI signal involving the corpus callosum, brainstem and cerebellum. COVID-19 may be associated with systematic modifications in brain morphometry and function. These alterations may be subtle, in particular at early stages of the disease process, and thus not evident by visual inspection alone. Such brain changes might be better captured by group-level statistical comparisons implemented in the following five case control studies.

Among the case control studies, Hosp et al. (2021) conducted a PET study looking at cerebral glucose metabolism in preselected COVID-19 patients with neurological symptoms. Results from a principal component analysis identified decreased metabolism in parietal and frontal association cortices in patients compared to controls. Conversely, patients exhibited lack of increased metabolism in brainstem, cerebellum, and mesial temporal lobe structures. Crunfli et al. (2021) investigated cortical abnormalities in non-hospitalised individuals diagnosed with COVID-19 relative to healthy volunteers. A surface-based morphometry method applied to brain MRI scans was used to identify group differences in the thickness of gray matter of the brain's surface. Individuals with COVID-19 showed decreased thickness in the olfactory sulcus, lingual gyrus, and calcarine sulcus and increased thickness in the central sulcus and superior occipital gyrus relative to the control group. The orbitofrontal alterations were attributed to the action of the virus in this cortical area, in line with a neuroinvasive mechanism of SARS-CoV-2 that uses the olfactory nerves as a gateway (Cooper et al., 2020).

Persisting brain changes throughout longer periods have been an additional focus of studies. Among the findings on individuals with recovered COVID-19, larger volumes of the olfactory cortex were shown compared to healthy volunteers (Lu et al., 2020). The study's MRI protocol included diffusion tensor imaging (DTI) to assess integrity of white matter. In addition to the olfactory alterations in gray matter concentration, individuals with COVID-19 had statistically larger volumes in hippocampi, insula, and cingulate gyrus and white matter alterations in corona radiata, external capsule and superior frontal-occipital fasciculus. Similarly, Donegani et al. (2021) performed PET scanning during recovery phase to investigate regional cerebral metabolism as well as integrity of connecting white matter tracts. Voxel-wise analysis shown hypometabolism in the parahippocampal, fusiform gyri, and insula in COVID-19 patients relative to healthy controls. The identified clusters were then subjected to structural connectivity analysis revealing involvement of the longitudinal fasciculus tract. The authors suggested that limbic hypometabolism encompassing the insula, is a potential disease substrate for recovered COVID-19 individuals while still experiencing olfactory dysfunction. Post-hospitalised COVID-19 patients were compared to comorbidity-matched healthy individuals by a brain MRI study assessing brain volume, white matter integrity and blood breakdown (e.g. haemorrhage) (Raman et al., 2021). Whereas no group differences were detected in brain volumes, COVID-19 patients had higher susceptibility-weighted imaging in the thalamus and disrupted white matter integrity in the sagittal stratum and posterior thalamic radiation. The authors associated their findings to higher burden of microvascular events among COVID-19 survivors. Additional abnormalities were found by Silva et al. (2021) reporting structural and functional brain MRI changes two months after the acute COVID-19 infection of non-hospitalised individuals. Structural changes in COVID-19 included increased diffusivity in the corpus callosum, corticospinal, dorsal cinguli, parahippocampal cinguli, uncinate fasciculi and inferior longitudinal fasciculi. In addition, functional connectivity analysis detected atypical connections in eleven networks. The highest number of altered connections was seen in the visuospatial network with 29 atypical connections affecting the superior parietal lobe/supramarginal gyrus/postcentral gyrus/angular gyrus.

Persisting brain changes were also shown in long-COVID by two PET studies (Sollini et al., 2021; Guedj et al., 2021). Sollini et al. (2021) found a hypometabolic pattern affecting the parahippocampal gyrus and thalamus in long-COVID patients compared to melanoma patients. Interestingly, COVID-19 patients with persistent anosmia/ageusia were characterised by hypometabolism in the parahippocampal gyrus and orbitofrontal cortex. Guedj et al. (2021) discovered hypometabolism in the rectal/orbital gyri, including the olfactory gyrus, and temporal lobe, including the amygdala and the hippocampus in patients manifesting complaints at least 3 weeks since COVID-19 infection. The hypometabolism of the frontal cluster, which included the olfactory gyrus, was worsened in patients taking angiotensin-converting enzyme (ACE) drugs for high blood pressure, and improved in those using nasal decongestant spray, favouring a role of ACE receptors as an olfactory gateway for this neurotropism.

The olfactory involvement was examined more closely by Niesen et al. (2021) on a combined MRI- PET study in COVID-19 patients with distortion of smell. Visual inspection of the MRI images revealed alterations of the olfactory cleft (i.e. inflammation and obliteration), suggesting that the virus reaches the brain via the olfactory nerves. The PET scans were subjected to voxel-based comparisons of regional cerebral glucose metabolism between the COVID-19 group and an independent healthy control dataset. COVID-19 patients presented decreases in glucose consumption in dorsolateral prefrontal, frontal eye fields, and anterior cingulate cortices, and metabolic rate increases in medial prefrontal cortex, posterior parietal cortex and thalamus. The authors interpreted that the alterations in high order areas underscored their involvement in the olfactory experience (i.e., strong emotions, memories and images, elicited by odours).

Throughout these studies a question emerges about differential brain changes between severe and mild COVID-19. Qin et al. (2021) investigated white matter microstructure and cerebral blood flow (CBF) 3-months from infection in patients with mild- and severe-type COVID-19 with no specific neurological manifestations or obvious lesions on the conventional MRI and totally recovered from pneumonia. The severe COVID group had reduced cortical thickness in limbic regions (e.g. insula, hippocampus and superior temporal gyrus). This patient group also showed greater decrease in CBF in frontal and temporal cortices than the healthy controls, with the insula being the region with the lowest CBF. Voxel-wise comparison on diffusion imaging, showed lower microstructure values in the severe COVID-19 group compared to the healthy volunteers across several white matter tracts, including the corticospinal tract and middle longitudinal fasciculus. Considering vulnerability to systemic inflammation during COVID-19, the authors explored associations between the brain measures and level of inflammatory markers. The brain's microstructural changes and CBF in the insula in the severe COVID-19 group were highly correlated with procalcitonin and interleukin-6 inflammatory markers.

Most compelling evidence for the olfactory involvement comes perhaps from a follow-up PET study (Kas et al., 2021) in patients with COVID-19. Patients manifested COVID-19 related encephalopathy and acute symptoms at baseline, clinical improvement within 2 months, and physical, and neurological recovery with normal autonomy at 6 months follow-up. Changes in regional cerebral glucose metabolism between patients and an independent healthy control dataset were assessed with a voxel-wise approach. At baseline COVID-19 patients had a pattern of hypometabolism involving the olfactory system (orbitofrontal cortex, and olfactory and rectus gyri) while spreading to the middle frontal gyrus, anterior cingulate, insula and hippocampi. Brain metabolism was progressively enhanced during follow-up, with first improvements at month 1 and only showing residual hypometabolism in the rectus/olfactory gyrus (with −9% decrease vs −19% at baseline), right insula (−10% vs −14%), anterior cingulate (−13% vs −20%), and middle frontal gyrus (−8% vs −20%) 6 months after COVID-19. An additional follow-up PET study identified frontoparietal and, to a lesser extent, temporal hypometabolism, which improved during six months after symptoms onset, in COVID-19 patients at subacute stage (Blazhenets et al., 2021). Duan et al. (2021), also conducting a follow-up study, investigated gray matter volume in patients with and without COVID-19. Changes in gray matter volume were identified through source-based morphometry analysis of computed tomography scans at both discharge and six-months follow-up phases. All patients showed lower gray matter volume in superior/medial/middle frontal gyri at both discharge and six months follow-up phases. However, patients with COVID-19 showed no significant differences in gray matter volume from patients without COVID-19 in any brain region.

Even though research on this area is scarce, preliminary findings do support some regional brain changes in mild and recovered cases. Seven out of the fourteen case control studies showed converging alterations in the central olfactory system affecting glucose metabolism (Kas et al., 2021; Guedj et al., 2021; Sollini et al., 2021), brain volume (Niesen et al., 2021; Lu et al., 2020), signal intensity (Strauss et al., 2020), and cortical thickness (Crunfli et al., 2021). Further consistent changes included functional and structural abnormalities in the insula and parahippocampus. However, it should be noted there are several inconsistencies between studies, with dispersed findings spanning across occipital, parietal, and temporal areas.

## 4 Discussion

This systematic review revealed abnormalities predominantly in the olfactory system (Coolen et al., 2020; Girardeau et al., 2020; Klironomos et al., 2020; Lin et al., 2020; Lu et al., 2020; Niesen et al., 2021; Crunfli et al., 2021; Kas et al., 2021; Aragao et al., 2021; Strauss et al., 2020; Blazhenets et al., 2021; Guedj et al., 2021; Sollini et al., 2021) and corpus callosum (Sawlani et al., 2020; Fitsiori et al., 2020; Chougar et al., 2020; Klironomos et al., 2020; Conklin et al., 2021; Dixon et al., 2020; Freeman et al., 2021) with additional consistent patterns involving insular (Kandemirli et al., 2020; Kas et al., 2021; Lu et al., 2020; Donegani et al., 2021; Qin et al., 2021) and prefrontal cortical regions (e.g. anterior cingulate: Kandemirli et al., 2020; Kas et al., 2021; Niesen et al., 2021; Girardeau et al., 2020; Lu et al., 2020) jointly implicating the *olfactory brain network*.

Disruptions of the olfactory sensory system in the present review affected four core structures: the olfactory epithelium, olfactory bulb, olfactory tract and primary olfactory cortices. The primary involvement of the olfactory system builds upon anosmia as an early marker of SARS-CoV-2 infection (Gerkin et al., 2020). These structural and functional manifestations may arise from systemic reactions such as coagulopathy, sepsis, autoimmune mechanisms or multiorgan failure (Deigendesch et al., 2020). Supporting this conceptualisation, Qin et al. (2021) found associations between white matter microstructure, and CBF in the insula correlated with procalcitonin and interleukin-6 inflammatory markers.

An alternative view argues that such regional brain changes may be due to direct viral brain invasion, causing for example, damage to the olfactory receptor neurons located in the olfactory epithelium. Notably, this review showed atrophy in this same olfactory region in individuals with COVID-19 (Niesen et al., 2021). In fact, compelling evidence from histological studies indicate high viral loads in the olfactory epithelium, where large amounts of SARS-CoV-2 entry proteins are expressed (Meinhardt et al., 2021).

Alterations of the olfactory system underscore the importance of its unique anatomical organisation that provides an environmental entry zone to the central nervous system (CNS). The olfactory nerves, in the peripheral nervous system, innervates the olfactory epithelium and terminates in the olfactory bulb in the CNS (Koyuncu et al., 2013). In this review SARS-CoV-2 was associated with structural (Coolen et al., 2020) and intensity abnormalities (Girardeau et al., 2020; Klironomos et al., 2020; Lin et al., 2020; Strauss et al., 2020; Aragao et al., 2021) in the olfactory bulb/tract (Table 3). These two structures are anatomically connected to the primary olfactory cortex (Patel and Pinto, 2014), which plays a critical role in the encoding of odorants (Kadohisa and Wilson, 2006). In the present findings, olfactory cortical deficits in SARS-CoV-2-infected individuals comprised altered cortical volume (Lu et al., 2020), thickness (Crunfli et al., 2021) and hypometabolism (Kas et al., 2021; Guedj et al., 2021; Sollini et al., 2021).

**Table 3:**
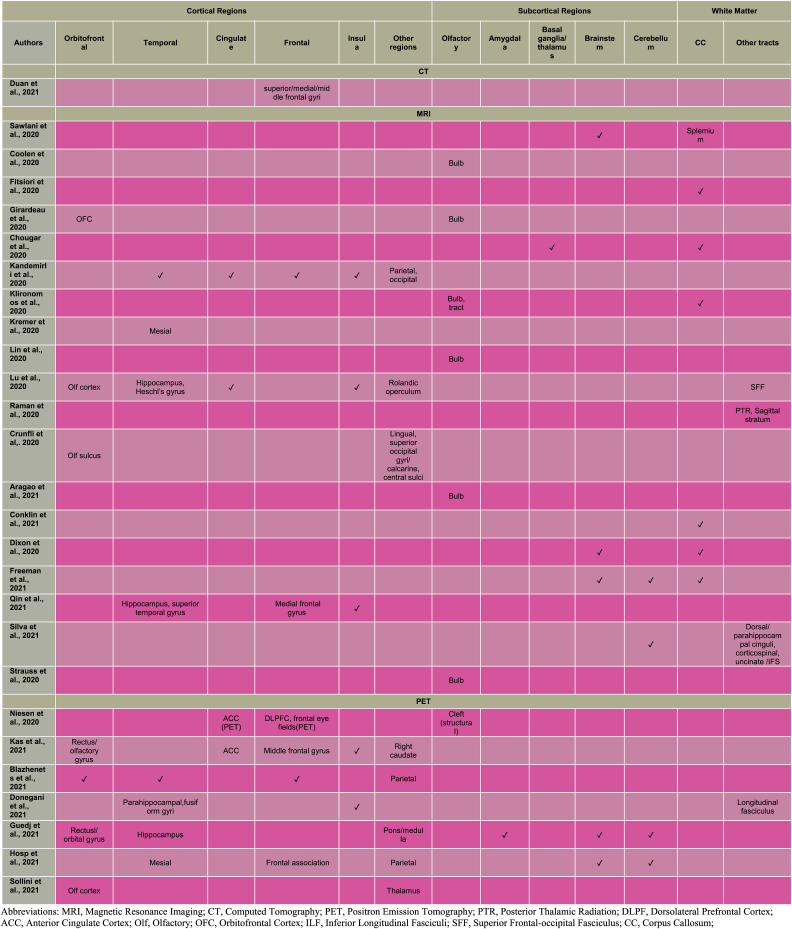
Implicated brain regions on SARS-CoV-2.

Beyond the olfactory system, olfactory cortices project to the orbitofrontal cortex, amygdala, hypothalamus, insula, entorhinal cortex, and hippocampus (Patel and Pinto, 2014). These projections may enable propagation of SARS-CoV-2 toward secondary olfactory areas. In fact, the present review showed alterations in several of these brain regions (Kandemirli et al., 2020; Kas et al., 2021; Lu et al., 2020; Niesen et al., 2021; Girardeau et al., 2020; Qin et al., 2021; Donegani et al., 2021; Guedj et al., 2021; Blazhenets et al., 2021; Sollini et al., 2021) (Table 3). The olfactory cortices contained in the temporal lobes, are inter-hemispherically connected via the corpus callosum (Zhou et al., 2013). This white matter structure is the largest commissural track, which integrates sensory information across the two cerebral hemispheres (Raybaud, 2010). A link between SARS-CoV-2 infection and changes in the corpus callosum was identified in this review with several studies showing consensus on structural abnormalities (Chougar et al., 2020; Conklin et al., 2021; Freeman et al., 2021) involving microbleeds (Fitsiori et al., 2020; Sawlani et al., 2020; Dixon et al., 2020). Alterations in a secondary olfactory cortex, the insula, including structural (Lu et al., 2020; Kandemirli et al., 2020) and functional changes (Kas et al., 2021; Donegani et al., 2021; Qin et al., 2021) were also associated with infection of SARS-CoV-2. The effects of the virus in the insula, a region specialised in processing smell and taste (Critchley et al., 2004), might undermine olfactory and gustatory functioning.

Likewise, higher order structures within the prefrontal cortex have been implicated in olfactory perception (for review see Savic, 2002). For example, an anterior cingulate functional pathway to the olfactory system is activated upon olfactory stimulation (Royet et al., 1999). The anterior cingulate, traditionally involved in attention processes (Mohanty et al., 2007) modulates olfactory perception instantiating olfactory experience. Alterations in the anterior cingulate cortex were also seen in SARS-CoV-2 infected individuals either exhibiting impaired connectivity (Lu et al., 2020), metabolism (Kas et al., 2021; Niesen et al., 2021) or signal intensity (Kandemirli et al., 2020). Based on the reviewed findings, SARS-CoV-2 infection could spread from the olfactory system to neighbouring brain regions, therefore encompassing the *olfactory brain network*; alternatively the structural and functional brain regional alterations may be due to systemic reactions to viral infection. The above discussion concentrates on common alteration patterns of brain regions across acute and mild severity groups. However, it should be noted that contrasting differences in nature and extent of localisation were also observed, with acute cases demonstrating more pronounced cerebral changes than mild and recovered cases. As mentioned in the results this might have been influenced by medical and neurological comorbidities, and intensive care, present in acute phases but less so in mild or recovered phases.

An alternative explanation of the brain events associated with SARS-CoV-2 proposes a mechanism initially targeting the brainstem and subsequently spreading towards the medullary cardiorespiratory center. This review found some support for this conceptualisation, with five studies detecting brainstem microbleeds (Sawlani et al., 2020; Dixon et al., 2020), altered intensity (Freeman et al., 2021), and hypometabolism (Guedj et al., 2021; Hosp et al., 2021) in individuals with SARS-CoV-2.

One important limitation in this review is associated with intrinsically methodological limitations of the included studies, namely lack of a control group (in 13 of 27 studies) and the cross-sectional design (24 studies). This review provided a qualitative synthesis of the data, therefore lacking a quantitative assessment of the literature. Also there is a need for meta-analysis; however, limited number of controlled neuroimaging studies on SARS-CoV-2, prevented us from carrying out such an approach. One other limitation is the exclusion of non-English language studies which may be significant considering that the virus spread internationally. Lastly, the present review included preprints to maximise the number of included studies, but at the same time including non-peer-reviewed articles could have introduced a source of bias. Beside these limitations, growing evidence of SARS-CoV-2 influence on the brain and the pressing need for increasing our understanding of the neural correlates of this viral infection, prompted us to review and broaden the inclusion criteria to case series and cohort studies.

## 5 Conclusion

In summary, brain alterations associated with SARS-CoV-2 infection in individuals with both acute and mild COVID-19, predominated in the olfactory brain network, which includes limbic and prefrontal structures. Whether disruptions in these brain regions were caused by direct or indirect viral infection remains uncertain; further longitudinal investigation -especially controlled studies, would be required to delineate the etiology of such neural deficits while potentially helping to identify impacted cognitive processes and elucidate longer-term effects.

## Funding and disclosure

This research did not receive any specific grant from funding agencies in the public, commercial, or not-for-profit sectors.

## Declaration of competing interest

The authors declare that they have no known competing financial interests or personal relationships that could have appeared to influence the work reported in this paper.
