# Supplementary material for: Brain imaging in patients with COVID-19: A systematic review
Source: Brain Behav Immun Health. 2021 Jul 2;16:100290. doi: 10.1016/j.bbih.2021.100290 (PMC8249107; doi:10.1016/j.bbih.2021.100290)
Supplement: Multimedia component 1 [file mmc1.docx]

Individual quality assessment of the included case-control studies

| Questions^a^ | Lu *et al* | Niesen *et al* | Raman *et al* | Crunfli *et al* | Kas *et al* | Blazhenets *et al* | Donegani *et al* | Duan *et al* | Guedj *et al* | Hosp *et al* | Qin *et al* | Silva *et al* | Sollini *et al* | Strauss *et al* |
| --- | --- | --- | --- | --- | --- | --- | --- | --- | --- | --- | --- | --- | --- | --- |
| 1-Were the criteria for inclusion in the sample clearly defined? | Yes | Yes | Yes | No | No | Yes | Yes | Yes | Yes | Yes | Yes | No | Yes | Yes |
| 2-Were the study subjects and the setting described in detail? | Yes | Yes | Yes | No | Yes | Yes | Yes | Yes | Yes | Yes | Yes | Yes | Yes | Yes |
| 3-Was the exposure measured in a valid and reliable way? | Yes | Yes | Yes | Yes | Yes | Yes | Yes | Yes | Yes | Yes | Yes | Yes | Yes | Yes |
| 4-Were objective, standard criteria used for measurement of the condition? | Yes | Yes | Yes | Yes | Yes | Yes | Yes | Yes | Yes | Yes | Yes | Yes | Yes | Yes |
| 5-Were confounding factors identified? | NA | Yes | NA | NA | Yes | Yes | Yes | Yes | NA | Yes | N/A | N/A | N/A | N/A |
| 6-Were strategies to deal with confounding factors stated? | NA | Yes | NA | NA | Yes | Yes | Yes | Yes | NA | Yes | N/A | N/A | N/A | N/A |
| 7-Were the outcomes measured in a valid and reliable way? | Yes | Yes | Yes | Yes | Yes | Yes | Yes | Yes | Yes | Yes | Yes | Yes | Yes | Yes |
| 8-Was appropriate statistical analysis used? | Yes | Yes | Yes | Yes | Yes | Yes | Yes | Yes | Yes | Yes | Yes | Yes | Yes | Yes |
| Total | 6/6 | 8/8 | 6/6 | 4/6 | 7/8 | 8/8 | 8/8 | 8/8 | 6/6 | 8/8 | 6/6 | 5/6 | 6/6 | 6/6 |

NA, not applicable

^a^According to the Joanna Briggs Institute (JBI) Critical Appraisal Checklist for Cross-sectional studies
