# Supplementary material for: Brain imaging in patients with COVID-19: A systematic review
Source: Brain Behav Immun Health. 2021 Jul 2;16:100290. doi: 10.1016/j.bbih.2021.100290 (PMC8249107; doi:10.1016/j.bbih.2021.100290)
Supplement: Multimedia component 2 [file mmc2.docx]

Individual quality assessment of the included case series

| Questions^a^ | Sawlani *et al* | Coolen *et al* | Fitsiori *et al* | Girardeau *et al* | Aragao *et al* |
| --- | --- | --- | --- | --- | --- |
| 1-Were there clear criteria for inclusion in the case series? | Yes | Yes | No | Yes | No |
| 2-Was the condition measured in a standard, reliable way for all participants included in the case series? | Yes | Yes | Yes | Yes | Yes |
| 3-Were valid methods used for identification of the condition for all participants included in the case series? | Yes | Yes | Yes | Yes | UN |
| 4-Did the case series have consecutive inclusion of participants? | Yes | Yes | No | Yes | No |
| 5-Did the case series have complete inclusion of participants? | Yes | Yes | UN | UN | Yes |
| 6-Was there clear reporting of the demographics of the participants in the study? | Yes | Yes | Yes | Yes | Yes |
| 7-Was there clear reporting of clinical information of the participants? | Yes | Yes | Yes | Yes | Yes |
| 8-Were the outcomes or follow up results of cases clearly reported? | Yes | Yes | Yes | Yes | Yes |
| 9-Was there clear reporting of the presenting site(s)/clinic(s) demographic information? | Yes | Yes | Yes | No | Yes |
| 10-Was statistical analysis appropriate? | Yes | Yes | UN | Yes | Yes |
| Total | 10/10 | 10/10 | 6/8 | 8/9 | 7/9 |

NA, not applicable; UN, unclear

^a^ According to the Joanna Briggs Institute (JBI) Critical Appraisal Checklist for Case Series
